# Pseudo2GO: A Graph-Based Deep Learning Method for Pseudogene Function Prediction by Borrowing Information From Coding Genes

**DOI:** 10.3389/fgene.2020.00807

**Published:** 2020-08-18

**Authors:** Kunjie Fan, Yan Zhang

**Affiliations:** ^1^Department of Biomedical Informatics, College of Medicine, The Ohio State University, Columbus, OH, United States; ^2^The Ohio State University Comprehensive Cancer Center, Columbus, OH, United States

**Keywords:** pseudogene, function prediction, graph neural networks, deep learning, gene ontology, feature propagation, semi-supervised learning

## Abstract

Pseudogenes are indicating more and more functional potentials recently, though historically were regarded as relics of evolution. Computational methods for predicting pseudogene functions on Gene Ontology is important for directing experimental discovery. However, no pseudogene-specific computational methods have been proposed to directly predict their Gene Ontology (GO) terms. The biggest challenge for pseudogene function prediction is the lack of enough features and functional annotations, making training a predictive model difficult. Considering the close functional similarity between pseudogenes and their parent coding genes that share great amount of DNA sequence, as well as that coding genes have rich annotations, we aim to predict pseudogene functions by borrowing information from coding genes in a graph-based way. Here we propose Pseudo2GO, a graph-based deep learning semi-supervised model for pseudogene function prediction. A sequence similarity graph is first constructed to connect pseudogenes and coding genes. Multiple features are incorporated into the model as the node attributes to enable the graph an attributed graph, including expression profiles, interactions with microRNAs, protein-protein interactions (PPIs), and genetic interactions. Graph convolutional networks are used to propagate node attributes across the graph to make classifications on pseudogenes. Comparing Pseudo2GO with other frameworks adapted from popular protein function prediction methods, we demonstrated that our method has achieved state-of-the-art performance, significantly outperforming other methods in terms of the M-AUPR metric.

## 1. Introduction

Pseudogenes were historically thought as unimportant DNA relics, since they have no protein-coding ability due to inactivating gene mutations during evolution (Vanin, 1985). However, more and more pseudogenes have been discovered to play important roles in gene regulation (Pink et al., 2011; An et al., 2017), especially in cancers (Xiao-Jie et al., 2015; Chan and Tay, 2018). One notable example is the transcriptional regulation of PTEN by pseudogene PTENP1 under several cancer conditions (Poliseno et al., 2010), indicating functional potentials of pseudogenes. With the accumulation of evidences showing the importance of pseudogenes, there has been renewed interest in the discovery of functional pseudogenes. Considering the huge amounts of existing pseudogenes, experimental validation of all their functions are time-consuming and expensive. Therefore, reliable computational methods to infer functions of pseudogenes are in great demand, which can be used to direct targeted experimental validation.

Several efforts have been made to study pseudogenes in a computational manner. Pseudofam is a large database of pseudogene families based on Pfam database, which can be used to analyze the family structure of pseudogenes (Lam et al., 2008). Han et al. (2014) proposed a supervised classification model to predict subtypes of endometrial cancer based on expression profiles of pseudogenes and highlights the prognostic power of pseudogenes. Johnson et al. (2018) adopted a novel graph-based approach to evaluate the relationship between pseudogenes and their parent genes. Pseudogene-gene (PGG) families are constructed based on sequence alignment and functional enrichment analysis can be performed in these families to infer functional impact of pseudogenes. PseudoFuN is a comprehensive PGG family database by taking advantage of the power of GPU computing (Johnson et al., 2019). However, there still remain several limitations in existing computational methods. First, all the above-mentioned methods only consider single features when studying pseudogenes, for example, DNA sequence or expression profile. The inclusion of multiple features might help characterize pseudogenes more comprehensively. Second, no computational methods have been proposed to directly infer functions of pseudogenes to guide biomedical researchers for targeted experimental validation. Gene Ontology (GO) is a comprehensive source of information on the functions of genes (Ashburner et al., 2000; Gene Ontology Consortium, 2018). A reliable machine learning model for predicting GO terms of pseudogenes is preferred.

Computational methods for predicting functions of coding genes (as well as proteins) have been studied for almost two decades. Most existing algorithms exploit homology inference to predict protein functions (Chitale et al., 2009; Loewenstein et al., 2009; Piovesan et al., 2011), based on the assumption that proteins with similar sequences tend to share similar functions. Some approaches involve the use of other features to infer protein functions, for example, protein-protein interaction (PPI) networks (Chua et al., 2007; Sharan et al., 2007), protein domains (Forslund and Sonnhammer, 2008; Rentzsch and Orengo, 2013), subcellular localization (Jensen et al., 2002; Lee et al., 2007), post-translational modifications (Jensen et al., 2002) and literature (Verspoor, 2014). Considering the limited capacity of a single feature source, many methods opt to combine multiple information and take advantage of the power of machine learning techniques. COFACTOR consists of three individual pipelines for sequence-, structure- and PPI-based predictions and generates the consensus based on three confidence scores obtained from three pipelines (Zhang et al., 2017). DeepGO uses representation learning methods to learn features from both sequence information and interaction networks respectively and then combine them to predict functions using a deep learning model (Kulmanov et al., 2017). Both Mashup (Cho et al., 2016) and DeepNF (Gligorijević et al., 2018) are network fusion methods for extracting integrated features from multiple heterogeneous interaction networks and then train a support vector machine (SVM) model to predict protein functions.

It is not suitable to directly apply protein function prediction algorithms to infer functions of pseudogenes, since functional annotations of pseudogenes is highly sparse, which is a significant challenge for traditional supervised machine learning methods. Semi-supervised learning is preferred in such a sparsely labeled setting. It is known that pseudogenes share similar functions with their parent genes based on homology inference (Johnson et al., 2018, 2019). Therefore, interaction networks between pseudogenes and coding genes can be constructed from the sequence similarity, where abundant labels of coding genes can be transferred to infer functions of pseudogenes. Besides the network information, the incorporation of more features is desirable to improve the robustness of the prediction model. Graph convolutional network (GCN) model is a neural network that operates on graphs and enables learning over graph structures, which was first proposed for semi-supervised classification (Kipf and Welling, 2016). The GCN model can naturally integrate both graph topology patterns and node features of graph data, and has significantly outperformed many state-of-the-art methods on several benchmarks (Wu et al., 2019). Due to its powerful capacity for representation and integration, it has been successfully applied in biomedical field that involves the use of graph data, including neuroimage analysis for Parkinson's Disease (Zhang et al., 2018), disease gene prioritization (Li et al., 2019), polypharmacy side effects prediction (Zitnik et al., 2018) and drug combination synergy prediction (Jiang et al., 2019).

In this work, we developed a multi-modal semi-supervised classification model based on GCN to predict functions of pseudogenes by considering multiple sources of information. Since each pseudogene may have multiple GO annotations simultaneously, this is a multi-label prediction model. We first build a similarity graph based on sequence similarity to connect pseudogenes and coding genes. For each node (pseudogenes, coding genes) in the graph, we consider expression profiles, interactions with microRNAs and node2vec embeddings of PPI and genetic interactions as node attributes (Grover and Leskovec, 2016), making the similarity graph an attributed graph. Then a two-layer GCN model is used to model this attributed graph, propagating node attributes across the graph. We compared our method with several state-of-the-art methods designed for protein function prediction in terms of three metrics. We have shown that Pseudo2GO outperforms all other methods in the comparison, demonstrating promising performance.

As far as we know, we are the first to propose a predictive model for inferring functions of pseudogenes on Gene Ontology directly. Our pseudogene-specific model is significantly better than those designed for protein function prediction when adapted for pseudogene function prediction. Besides, our model is extensible to incorporate more features as node attributes to further improve the performance. The satisfying performance of Pseudo2GO makes it desirable to be used for screening functional pseudogenes for experimental validation.

## 2. Materials and Methods

### 2.1. Data Collection

Human pseudogene and protein coding gene annotations were obtained from GENCODE release 29 (Frankish et al., 2018). We only consider transcribed pseudogenes in our analysis as they possess greater functional potentials. We collected two groups of gene expression profiles: median expression values per tissue from GTEx V8 (Lonsdale et al., 2013) and BRCA expression values from dreamBase (Zheng et al., 2017), a large-scale database for pseudogenes. For GTEx expression data, TPM median expression values for all 54 tissues are used to characterize each gene. The BRCA expression values are from TCGA database and curated by dreamBase, and we will refer it as TCGA expression feature. Genetic interactions and protein-protein interactions (PPI) were downloaded from BioGRID version 3.5.173 (Oughtred et al., 2018) and microRNA-target interactions (MTI) were downloaded from miRTarBase release 7.0 (Chou et al., 2017). We used Gene Ontology terms as the functional annotation that were download from Gene Ontology knowledgebase (release 2019-03-19).

### 2.2. Data Preprocessing and Encoding

**Figure 1:**
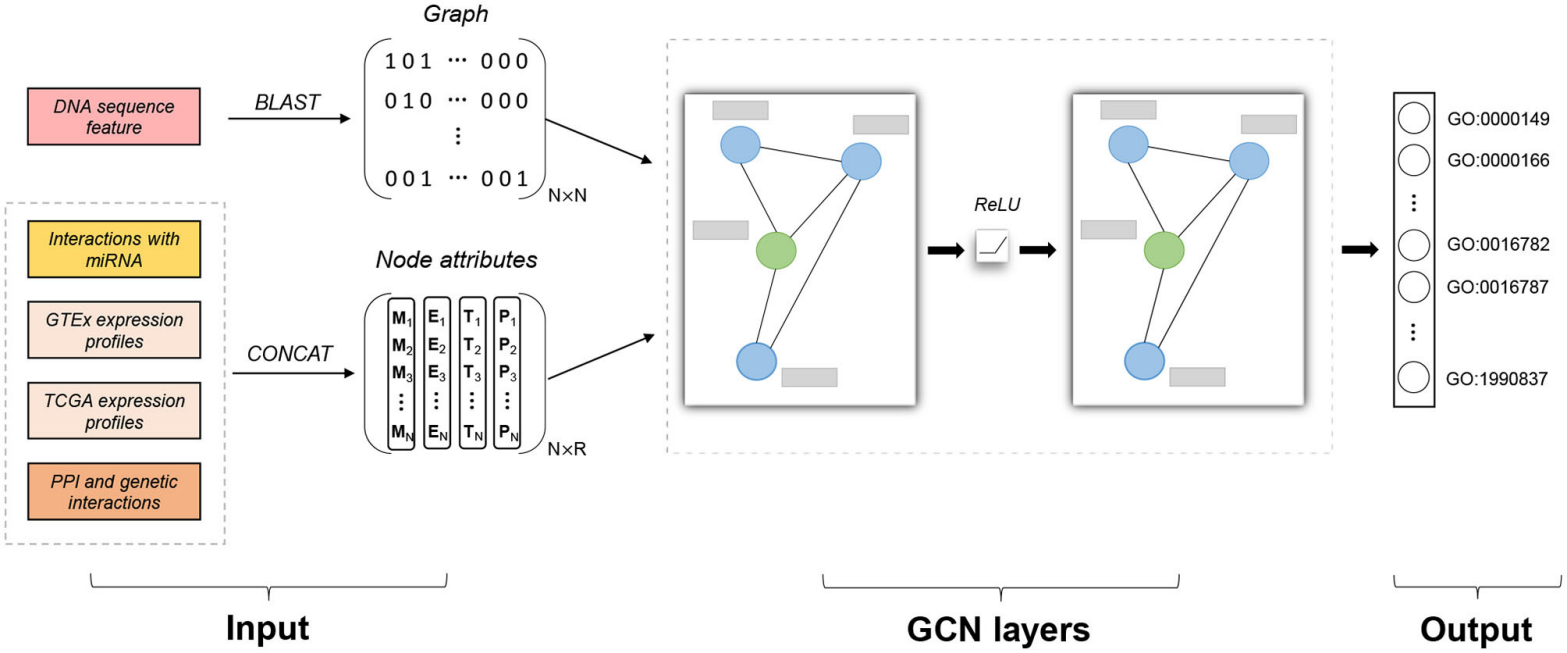
Model architecture of Pseudo2GO. There are two types of input features: sequence similarity network (graph information) and node attributes (including four kinds of features). Similarity graph is obtained by running BLAST on DNA sequence features. Four kinds of node attributes are first encoded and then concatenated together to form the final node attribute matrix X with the dimension of *N* × *R*. Two layers of graph convolutional network (GCN) are stacked to process the attributed graph. The green node in the graph refers to a pseudogene, while the blue ones are coding genes. The gray bar represents node attribute vector for each gene (pseudogene or coding gene). The output is a set of GO terms we are going to predict (multi-label prediction). Our model gives a probability score for each GO term. Adam algorithm is used to train the model by minimizing the cross entropy error. Our model is a semi-supervised machine learning model, training on coding genes while testing on pseudogenes.

There are two kinds of features in our model: similarity graph and node attributes, which are integrated to make classifications, as shown in Figure 1. The graph represents the structural information of data, while each data instance also comes with feature vectors containing important information not present in the graph. We first discuss how to construct the similarity graph and then how to encode informative features as node attributes.

#### 2.2.1. Graph Construction

In order to infer pseudogene functions by borrowing information from coding genes, the first step is to construct a similarity graph connecting these two kinds of genes. Considering that pseudogenes have high sequence similarity with coding genes, especially with their parent coding genes that share similar functional annotations, constructing a graph based on sequence similarity can help build the relationship between pseudogenes and coding genes in the functional domain.

BLAST is used to detect similar gene pairs based on sequence similarity (Altschul et al., 1990). As we can see from Figure S1, there are a large portion of highly similar gene pairs whose e-value equals to zero. In order to make the selected edges of high confidence, we set the threshold as 1e-200 and only keep those pairs whose e-value are less than 1e-200 to construct the graph. In our dataset, we only select coding genes which share high sequence similarity with at least one pseudogene, resulting in 7,527 coding genes and 1,151 transcribed pseudogenes left. After filtering, there are 2,865,136 edges in the graph, where pseudogenes involve in 156,582 interactions.

#### 2.2.2. Node Attributes

There are in total four kinds of node attributes used in our model: two groups of expression profiles, PPI and genetic interactions, and interactions with microRNAs. By including PPI and genetic interactions in our model, we characterize the relationship between pseudogenes and coding genes more comprehensively. We take into account the interactions with microRNAs, whose importance have been implicated in competing endogenous RNA (ceRNA) networks, where pseudogenes act as decoy targets for microRNAs targeting protein-coding genes (Salmena et al., 2011). It is possible that pseudogenes and coding genes sharing the same microRNAs may be involved in the same cellular mechanisms and thus share similar functional annotations (Poliseno and Pandolfi, 2015).

The way of encoding these node attributes greatly affects the performance of our GCN model. For interactions with microRNAs, we only consider microRNAs that have more than 250 targets in the database, resulting in 118 microRNAs left. For each gene, we use bag-of-words encoding to represent this information. The encoding vector is of length 118 consisting of 0's and 1's, where 1 means the gene interacts with the corresponding microRNA and 0 otherwise.

As for other three types of node attributes, we adopt a learning-based method for encoding as suggested by Duong et al. in their study on node attributes for graph neural networks (Duong et al., 2019). For both GTEx and TCGA expression data, we first calculate the Spearman correlation and select pairs whose correlation are higher than 0.5 or less than −0.5 to build the co-expression network. Then node2vec algorithm is applied on these co-expression networks to generate embeddings for each node (gene) (Grover and Leskovec, 2016). These embeddings represent global structure information contained in the co-expression patterns, which can be used to differentiate genes and make classification, and we have shown that they are more informative than raw expression values. As for PPI and genetic interactions, we repeat the same procedure to generate latent embeddings by node2vec to represent topology information within the network. The length of the embeddings for all above three attributes is 256.

### 2.3. Problem Setting

We are given an undirected graph $G=\left(V_{p},V_{c},E\right)$ where $V_{p}$ are pseudogene nodes and $V_{c}$ are coding gene nodes, with $N_{p}=|V_{p}|$, $N_{c}=|V_{c}|$ and *N* = *N*_*p*_ + *N*_*c*_. The adjacency matrix A of G and its diagonal degree matrix D are derived from known graph information, where each edge is a similarity pair in the sequence similarity graph. Four kinds of node attributes are represented as E (expression profiles from GTEx dataset), T (expression profiles from TCGA database), M (interactions with microRNAs) and P (PPI and genetic interactions) with the dimension of *N* × 256, *N* × 256, *N* × 118 and *N* × 256. These four matrices are concatenated into one final node attribute matrix X with the dimension of *N* × *R*, where *R* equals 886.

Since Gene Ontology (GO) has three categories—cellular component (CC), molecular function (MF), and biological process (BP), we use Y_*cc*_, Y_*mf*_, and Y_*bp*_ to denote them separately. They are label indicator matrices consisting of 0's and 1's. For GO annotations, we only consider the experimental evidence code among EXP, IDA, IPI, IMP, IGI, IEP, TAS, and IC. (Note: Guide to GO Evidence Codes http://www-legacy.geneontology.org/GO.evidence.shtml). If a gene is annotated with a GO term, we additionally annotated it with all the ancestor terms. Due to the small number of annotations for very specific GO terms, we rank GO terms by their number of occurrences and select the top 339 terms for CC, 368 terms for MF and 309 terms for BP. The corresponding cutoff values for selecting these terms are 25, 25, and 250 for CC, MF, and BP, respectively.

Our goal is to predict pseudogene functions on CC, MF, and BP separately by training the model using coding genes, in a graph-based semi-supervised manner. Considering that coding genes have rich annotations, we try to maximize the effective utilization of structural and feature information of well-studied coding genes by using graph convolutional networks.

### 2.4. Graph Convolution

Traditional convolutional neural networks (CNN) rely on the regular grid-like structure with a well-defined neighborhood (Krizhevsky et al., 2012). However, for a graph structure, there is no natural choice for an ordering of the neighbors of a node, therefore the convolution operation needs to be adapted. Given an undirected graph with node attribute matrix X and adjacency matrix A, the graph convolution operation is defined as:

(1) $$\begin{array}{l} \text{H}=f\left({\hat{\text{D}}}^{-\frac{1}{2}}\hat{\text{A}}{\hat{\text{D}}}^{-\frac{1}{2}}\text{XW}\right)\text{,} \end{array}$$

where $\hat{\text{A}}=\text{A}+\text{I}$, I is the identity matrix, ${\hat{\text{D}}}_{ii}=\underset{j}{\sum }{\hat{\text{A}}}_{ij}$, W is the trainable weight matrix for neural network, H is the updated feature matrix and f is the activation function, e.g., ReLU(·) = max(0, ·).

Intuitively, this graph convolution operation computes the new features of a node as the weighted average of node attributes of itself and its neighbors', similar to Laplacian smoothing which makes features of nodes in the same cluster similar (Li et al., 2018). This operation naturally combines both graph structures and node attributes in the convolution, where the features of unlabeled nodes (pseudogenes in our case) are mixed with those of nearby labeled nodes (coding genes), and propagated over the graph structure. By this aggregation scheme, intuitively, if two nodes have identical neighboring structures with identical node features on the corresponding nodes, their embeddings H will be exactly identical (Xu et al., 2018). In other words, the embeddings are a good characterization to measure similarities based on both graph information and node features, and thus promising to be used for classification.

### 2.5. Pseudo2GO

The graph convolution operation can be stacked into multiple layers to enable learning over a larger neighborhood structure. However, a GCN model with too many layers is not a good choice since repeatedly applying Laplacian smoothing may mix the features of nodes from different clusters and make them indistinguishable (Li et al., 2018). Here we adopted a two-layer model suggested in Li et al. (2018) and Kipf and Welling (2016), as shown in Figure 1. Our Pseudo2GO model is defined as:

(2) $$\begin{array}{l} \text{Z}=\sigma \left(\tilde{\text{A}}\mathrm{ReLU}\left(\tilde{\text{A}}\text{X}{\text{W}}^{0}\right){\text{W}}^{1}\right)\text{.} \end{array}$$

where $\tilde{\text{A}}={\hat{\text{D}}}^{-\frac{1}{2}}\hat{\text{A}}{\hat{\text{D}}}^{-\frac{1}{2}}$, σ is the sigmoid function, W^0^ and W^1^ are both trainable weight matrices. Here $\tilde{\text{A}}$ is the symmetrically normalized adjacency matrix in order to avoid changing the scale of the feature vectors X.

The loss function is defined as the binary cross entropy error over all coding genes:

(3) $$\begin{array}{l} L=-\underset{i\in V_{c}}{\sum }\overset{F}{\underset{f=1}{\sum }}{\text{Y}}_{if}\ln{\text{Z}}_{if}\text{.} \end{array}$$

where $V_{c}$ is the set of indices of coding gene nodes and F is the column dimension of the output matrix, which is equal to the number of labels (GO terms) in this multi-label setting. The Model is trained using stochastic gradient descent by updating weight matrices so as to minimize loss function.

## 3. Results

### 3.1. Experimental Setup

Pseudo2GO was implemented using PyTorch Geometric library in Python and took advantage of the powerful computing capacity of GPU (Fey and Lenssen, 2019). All the simulations were carried out on Owens cluster provided by the Ohio Supercomputer Center (OSC) with 27 processors and 127GB memory (Ohio Supercomputer Center, 1987). The GPU we used was NVIDIA Tesla P100 with 16GB memory. Our source code is available at https://github.com/yanzhanglab/Pseudo2GO. In our dataset, there are in total 7,527 coding genes and 1,151 transcribed pseudogenes. Coding genes are used as the training set while pseudogenes are in our test set used for evaluation. Several hyper-parameters need to be determined: number of neurons of the hidden layer, learning rate and number of training iterations. 5-fold cross-validation was performed on the training data to select the best hyper-parameters. We end up with choosing 256 as the number of units in the hidden layer. The number of units in the output layer of our model equals the number of GO terms in CC, MF or BP ontology. The model is trained for 400 iterations using Adam algorithm with a learning rate of 0.01 (Kingma and Ba, 2014).

We used three evaluation metrics for this multi-label task: the macro-averaged area under the precision-recall curve (M-AUPR), the micro-averaged area under the precision-recall curve (m-AUPR) and the harmonic mean of precision and recall when the top three predictions are assigned to each gene (F1-score). The formal definition of F1-score is as follows:

(4) $$\begin{array}{l} pr\left(t\right)=\frac{\sum_{i}\sum_{f}I\left(f\in P_{i}\left(t\right)\wedge f\in T_{i}\right)}{\sum_{i}\sum_{f}I\left(f\in P_{i}\left(t\right)\right)}\text{,} \end{array}$$

(5) $$\begin{array}{l} rc\left(t\right)=\frac{\sum_{i}\sum_{f}I\left(f\in P_{i}\left(t\right)\wedge f\in T_{i}\right)}{\sum_{i}\sum_{f}I\left(f\in T_{i}\right)}\text{,} \end{array}$$

(6) $$\begin{array}{l} F_{1}\left(t\right)=\frac{2\cdot pr\left(t\right)\cdot rc\left(t\right)}{pr\left(t\right)+rc\left(t\right)}\text{.} \end{array}$$

where *pr* means precision, *rc* means recall, *I* is the indicator function, f is a GO term, *P*_*i*_(*t*) is a set of predicted GO terms for gene *i* using the threshold *t*, and *T*_*i*_ is a set of annotated GO terms for gene *i*. In our implementation of F1-score, in order not to determine a threshold, we only consider the top three predictions and calculate the F1-score. This implementation is also utilized by Mashup (Cho et al., 2016) and DeepNF (Gligorijević et al., 2018).

The other two evaluation metrics M-AUPR and m-AUPR are widely used when the labels are highly imbalanced, and it has been proved that AUPR is more informative than area under the receiver operating characteristic curve (ROC-AUC) in the imbalanced case (Davis and Goadrich, 2006). The formal definition of these two metrics is as follows:

(7) $$\begin{array}{l} pr_{f}\left(t\right)=\frac{\sum_{i}I\left(f\in P_{i}\left(t\right)\wedge f\in T_{i}\right)}{\sum_{i}I\left(f\in P_{i}\left(t\right)\right)}\text{,} \end{array}$$

(8) $$\begin{array}{l} rc_{f}\left(t\right)=\frac{\sum_{i}I\left(f\in P_{i}\left(t\right)\wedge f\in T_{i}\right)}{\sum_{i}I\left(f\in T_{i}\right)}\text{,} \end{array}$$

(9) $$\begin{array}{l} {\text{AUPR}}_{f}=\underset{t}{\sum }\left(rc_{f}\left(t\right)-rc_{f}\left(t-1\right)\right)\cdot pr_{f}\left(t\right)\text{,} \end{array}$$

(10) $$\begin{array}{l} \text{M-AUPR}=\frac{1}{N_{f}}\cdot \underset{f}{\sum }{\text{AUPR}}_{f}\text{.} \end{array}$$

(11) $$\begin{array}{l} \text{m-AUPR}=\underset{t}{\sum }\left(rc\left(t\right)-rc\left(t-1\right)\right)\cdot pr\left(t\right)\text{.} \end{array}$$

where *pr*_*f*_ and *rc*_*f*_ are precision and recall for a single GO term f, AUPR_*f*_ is the area under the precision-recall curve (AUPR) for f, *N*_*f*_ is the number of GO terms used for evaluation. The macro-averaged AUPR (M-AUPR) is defined as the unweighted mean of the AUPR for all labels, while the micro-averaged AUPR (m-AUPR) is calculated globally by considering each element of the label indicator matrix as a label.

### 3.2. Integration of Multiple Node Attributes Improves the Performance

In our Pseudo2GO model, we use four kinds of features as node attributes, as mentioned before. Here, we train one individual model for each attribute to demonstrate the power of integration. For each individual model, we use the same graph information, training on the same training set (coding genes) and testing on pseudogenes. The only difference between these models is the choice of node attribute. Simulations were repeated 10 times for each model and bootstrap was used to estimate the confidence interval.

As shown in Table 1, the model that includes all four kinds of features greatly outperforms other individual models that only use one feature as node attribute, demonstrating the importance of integrating multiple features. Looking at these four individual models, we can see that the two types of expression are the most informative features, achieving the best performance in terms of M-AUPR and F1-score on both CC and BP. Besides, the model based on PPI and genetic interactions achieves the highest M-AUPR score on MF ontology. Among all four individual models, the model using interactions with microRNA as the node attribute works the worst. This might be due to the sparse encoding which makes training hard.

**Table 1:** Comparison between different node attributes.

**Node attribute**	**CC**		**MF**		**BP**	
	**M-AUPR**	**F1-score**	**M-AUPR**	**F1-score**	**M-AUPR**	**F1-score**
microRNA	0.292 ± 0.02	0.357 ± 0.01	0.211 ± 0.05	0.263 ± 0.01	0.230 ± 0.03	0.192 ± 0.01
PPI	0.415 ± 0.03	0.369 ± 0.01	0.346 ± 0.02	0.291 ± 0.01	0.264 ± 0.02	0.191 ± 0.02
TCGA-exp	0.462 ± 0.07	0.376 ± 0.01	0.319 ± 0.03	0.278 ± 0.01	0.338 ± 0.02	0.184 ± 0.01
GTEx-exp	0.462 ± 0.09	0.373 ± 0.01	0.271 ± 0.03	0.301 ± 0.01	0.308 ± 0.04	0.195 ± 0.01
GTEx-raw	0.325 ± 0.02	0.357 ± 0.01	0.224 ± 0.02	0.257 ± 0.01	0.211 ± 0.02	0.183 ± 0.01
shuffle	0.463 ± 0.10	0.376 ± 0.02	0.385 ± 0.09	0.316 ± 0.02	0.306 ± 0.06	0.185 ± 0.01
ALL	0.587 ± 0.02	0.380 ± 0.01	0.463 ± 0.02	0.319 ± 0.01	0.362 ± 0.01	0.193 ± 0.01

*M-AUPR and F1-score are used for evaluation. PPI represents PPI and genetic interactions. TCGA-exp and GTEx-exp stand for expression profiles from TCGA and GTEx that are processed by node2vec. GTEx-raw represents raw expression values from GTEx. ALL is our final model that includes all four kinds of features as the node attributes*.

We also tested the model performance when using different combinations of node attributes, as shown in Table S1. The results are consistent with Table 1. In CC and BP ontology, since two types of expression features are the most informative, combining these two features only can achieve impressive performance, even slightly outperforming the model using all four kinds of node attributes. As for MF ontology, since PPI and genetic interactions are also informative, the model using two types of expression as well as PPI achieves outstanding performance, but not as good as the model with all node attributes.

### 3.3. Learning-Based Encoding for Node Attributes Is Better Than Raw Information

As suggested by Duong et al. in their research on node attributes for graph neural network models (Duong et al., 2019), learning-based method for encoding node attributes can improve the model performance. In our model, for both two types of expression features and PPI and genetic interactions feature, we transform their raw representations into learned embeddings by applying node2vec algorithm (Grover and Leskovec, 2016). It should be noted that for two types of expression features, co-expression network should be constructed first in order to run node2vec. node2vec is a representation learning method where continuous low-dimensional representations for nodes in the graph can be learned by optimizing a neighborhood preserving objective.

In order to show the learning-based encoding is better than raw representation, we compared the models that use raw GTEx expression profiles or GTEx expression feature after node2vec processing as node attribute. As shown in Table 1, the model using learning-based feature achieves significantly better performance than the one using raw feature as the node attribute, especially in terms of M-AUPR. When we feed raw feature into the model, considering that the data may be of low quality or contains some noises, the entire load of learning is put on the model, making it hard to train and generalize. On the contrary, we already put some knowledge into the data by applying node2vec to learn informative representations, making it of high quality and easier for the model to learn.

### 3.4. Graph Information Is Important for Pseudogene Function Prediction

**Figure 2:**
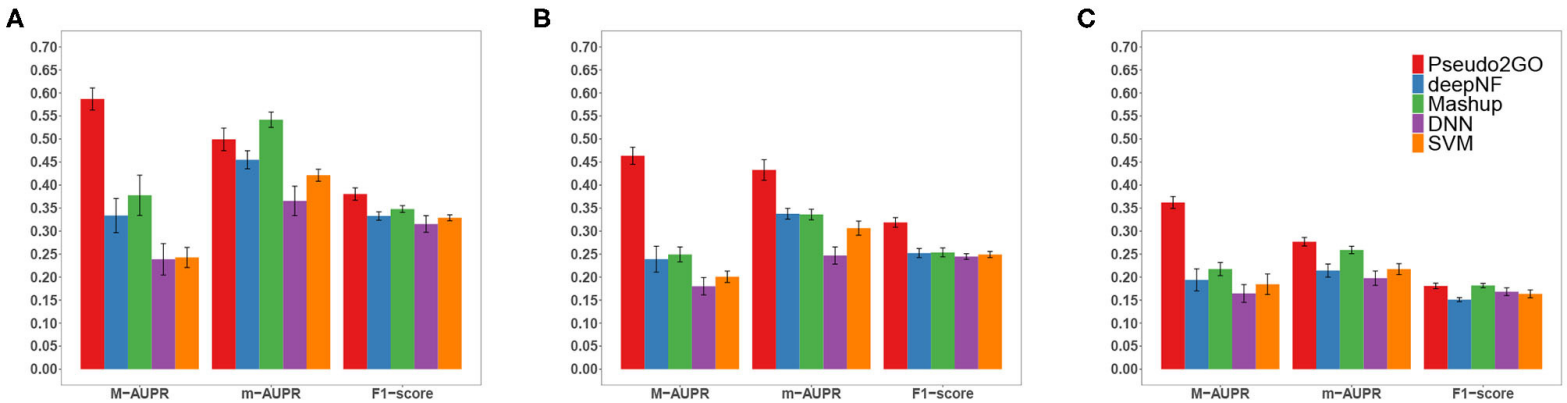
Performance comparison of Pseudo2GO with other machine learning methods. Macro-AUPR, micro-AUPR and F1-score are used for evaluating the performance. Panels **(A–C)** show the comparison results on CC, MF and BP ontology, respectively. Each method is evaluated using 5-fold cross-validation, repeated 10 times to calculate the confidence interval.

In order to show the importance of using graph information to borrow information from coding genes based on GCN model to predict functions of pseudogenes, we shuffle the node attributes and evaluate the performance. As we can see from Table 1, even with the completely randomized features, the model can still achieve a reasonable performance, comparable to the individual model using expression feature as node attribute. This can be attributed to the power of using graph information and GCN model that makes features of nodes in the same cluster similar, which helps subsequent classification. If we look at the performance of SVM and DNN models shown in Figure 2, we can see that our method outperforms them by a large margin. These two models use the same node attributes (four kinds of features) in our method as features, which means the only difference between them and our method about features is whether to use graph information to transfer knowledge. It is indicated that only using node features without graph information is not desirable, further demonstrating the importance and necessity of using graph information.

### 3.5. Pseudo2GO Outperforms Other Machine Learning Methods

We have shown that both graph information and node attributes are informative and important for predicting functions of pseudogenes. In order to show the superiority of our method Pseudo2GO, we compare it with four other machine learning methods. deepNF (Gligorijević et al., 2018) and Mashup (Cho et al., 2016) are two state-of-the-art network fusion methods for protein function prediction. Sequence similarity network, co-expression network and PPI network features are used in these two methods. We also compare it with two machine learning models that are not based on graph information: support vector machine (SVM) and deep neural network (DNN), which use the same node attributes used in our method as the input features. For above-mentioned four methods, we also use 5-fold cross-validation on training data to choose hyper-parameters.

It is shown that our method achieves the best performance on all three ontologies in terms of all metrics, except that Mashup outperforms our method on CC in terms of m-AUPR. It should be noted that in terms of M-AUPR, Pseudo2GO outperforms other four methods by a large margin (about 0.2 higher than the second best method on all three ontologies), showing the superiority of our method. We can also see that on MF ontology, our method significantly outperforms other methods in terms of all three metrics, indicating that the use of graph information about sequence similarity is highly informative for predicting molecular function (MF). Among all five methods, the SVM and DNN model work the worst, though they are among the most popular methods in predicting protein functions. Given the limited annotation of existing pseudogenes, without borrowing information from well-studied coding genes by way of graphs, these two models are not able to learn sufficiently good classifiers. Comparing our method with Mashup and deepNF, two network fusion models, the inclusion of more features (node attributes) and the powerful representation capacity of GCN make Pseudo2GO a much better model.

### 3.6. Pseudo2GO Shows Better Precision Than BLAST for Inferring Pseudogene Functions

It is widely known that pseudogenes exhibit great DNA sequence similarity with their parent coding genes, resulting from the inactivating gene mutations during evolution. The similarity in DNA sequence implicates the similarity in functions, for example, pseudogenes act as decoy targets for microRNAs that target protein-coding genes because of the same microRNA response elements, forming the competing endogenous RNAs (ceRNAs) and showing almost the same functions. When analyzing the functional relationship between pseudogenes and their parent genes, out of limited pseudogenes with functional annotations (only 97 pseudogenes have GO term annotations on MF ontology), we found out 10 pairs of pseudogene-coding gene have exactly the same GO terms annotation on MF ontology. These pairs include FKBP9P1-FKBP9, CA5BP1-CA5B, DPY19L2P1-DPY19L2, CES1P1-CES1, TDGF1P3-TDGF1, STAG3L1-STAG3, STAG3L2-STAG3, STAG3L3-STAG3, and STAG3L4-STAG3. These evidences show that transferring functional annotations of the parent coding gene to the corresponding pseudogene can be an effective method.

BLAST is a sequence alignment tool that can be used to search the most similar gene for the query pseudogene (Altschul et al., 1990). For each pseudogene, BLAST program is used to search against all coding genes to select the most similar one (probably the parent gene), and then assign all functions of this target gene to the query pseudogene as the prediction. We compared our method with this BLAST-based method. F1-score (harmonic mean between precision and recall), precision and recall metrics are used for evaluation. In order to calculate these metrics for our method, we choose the threshold (between 0 and 1) for each ontology such that the F1-score is maximized (Radivojac et al., 2013). As shown in Table S2, in terms of F1-score, our method is better than BLAST on CC and BP, but slightly worse on MF. Looking at the precision, our method shows promising results, a lot higher than BLAST. By directly borrowing functional annotation from the most similar gene, BLAST can achieve high recall score, as we showed previously that there is a large correlation between sequence similarity and functions. However, this method is not very accurate and robust, resulting in many false positives, given that only sequence information is considered. Compared to BLAST, our method not only borrows information from multiple similar genes by constructing a network, but also considers several node attributes, making it more comprehensive and robust.

In order to further show the promising performance of our method in predicting novel functions, for each pseudogene, we sorted the prediction scores across all GO terms and selected the top 5 predictions with the highest confidence. Then we calculated the proportion of these 5 predictions belonging to the true annotations. Out of 97 pseudogenes with at least one true MF annotations, 31 (31.9%) pseudogenes got 100% proportion. There were 46 (49.5%) out of 93 pseudogenes got 100% proportion in CC ontology, and 30 (34.5%) out of 87 in BP ontology. In Table S3, we listed selected 9 pseudogenes where our model's top 5 prediction were all true positives across all three ontologies. As we can see, the top predictions of our method are reliable and can be used for inferring novel functions not present in the database.

## 4. Discussion

Pseudo2GO is a graph-based deep learning model for predicting functions of pseudogenes by borrowing information from coding genes. DNA sequence similarity information is used to build a graph connecting pseudogenes and coding genes, where multiple features are incorporated to characterize each node (gene). This attributed graph is modeled by a two-layer graph convolutional network which is capable of capturing both graph structural information and node attributes. We are the first to directly predict pseudogene functions on Gene Ontology, which can help guide the experimental validation. Comparing our method to other popular methods designed for protein function prediction, Pseudo2GO has achieved state-of-the-art performance.

One significant challenge for predicting pseudogene functions is the huge amount of missing features and functional annotations, making traditional supervised learning models inapplicable. Our model managed to solve this problem in several ways. First, as coding genes have plentiful features, putting pseudogenes and coding genes in the same pool by building a similarity graph helps pseudognes borrow information from coding genes using GCN model. Second, considering only limited pseudogenes have functional annotations, incorporating coding genes into our model can be regarded as a way to increase the sample size, which is important for training a deep learning model. Third, when encoding node attributes with lots of missing values, node2vec algorithm helps generate more informative representations. For expression data from TCGA, there are more than 50% missing values for genes used in our dataset, which can not be directly encoded. After constructing the co-expression network and using node2vec to process the network, the newly generated representations are free of missing values and provide informative features.

Since the graph is constructed based on sequence similarity, it is possible that several protein coding genes of the same paralog families connect to one pseudogene simultaneously, as shown in Figure S2 (using pseudogene AC114812.1 as an example). Since node attributes and labels of coding genes belonging to the same paralog family tend to be clustered together, when the pseudogene borrows information from neighboring coding genes, it can be regarded that multiple copies of the similar node attributes will be used to enrich the pseudogene. The problem is that the learning of the pseudogene feature may be biased to the paralog family with lots of instances. In the future, we may consider adding edge weight for each similarity pair and normalizing the weight for edges connecting with coding genes of the same paralog family to solve this potential problem.

Regarding to the future direction, our model has the potential to be further improved. Currently, we only utilize one kind of graph information (sequence similarity network) to connect pseudogenes and coding genes. To fully take advantage of the power of GCN, building a heterogeneous network consisting of pseudogenes, coding genes, microRNAs and maybe lncRNAs may worth a try in the future, because this heterogeneous network characterizes a more comprehensive relationship between pseudogenes and other kinds of genes or RNAs. Besides, node attributes defined in our model can be easily extended to incorporate more discriminating features to improve the performance. We can also relax the criterion for building interactions between pseudogenes and coding genes. For example, instead of calculating the similarity based on the whole sequences, we can only focus on certain intact domains to measure the similarity.

## Data Availability Statement

Our source code is available at https://github.com/yanzhanglab/Pseudo2GO.

## Author Contributions

KF developed the software, YZ conceived and supervised the project. KF and YZ wrote the manuscript and approved it for publication.

## Conflict of Interest

The authors declare that the research was conducted in the absence of any commercial or financial relationships that could be construed as a potential conflict of interest.
