# Supplementary material for: Pseudo2GO: A Graph-Based Deep Learning Method for Pseudogene Function Prediction by Borrowing Information From Coding Genes
Source: Front Genet. 2020 Aug 18;11:807. doi: 10.3389/fgene.2020.00807 (PMC7461887; doi:10.3389/fgene.2020.00807)
Supplement: Supplementary file 1 [file Data_Sheet_1.DOCX]

Supplementary Materials

**Supplementary tables**

**Table S1**. Performance comparison among combinations of different node attributes. All combinations of two node attributes and three node attributes are compared. In this table, “M” stands for “microRNA”, “P” as “PPI”, “T” as “TCGA-expression” and “E” as “GTEx-expression” feature.

|  | CC | | MF | | BP | |
| --- | --- | --- | --- | --- | --- | --- |
| Node attribute | M-AUPR | F1-score | M-AUPR | F1-score | M-AUPR | F1-score |
| M+P | 0.439±0.02 | 0.382±0.01 | 0.392±0.01 | 0.301±0.01 | 0.289±0.02 | 0.195±0.01 |
| M+T | 0.549±0.11 | 0.380±0.01 | 0.378±0.03 | 0.293±0.01 | 0.364±0.01 | 0.191±0.01 |
| M+E | 0.437±0.11 | 0.374±0.02 | 0.304±0.05 | 0.308±0.01 | 0.322±0.02 | 0.195±0.01 |
| P+T | 0.564±0.02 | 0.392±0.01 | **0.425±0.02** | 0.302±0.01 | 0.361±0.01 | 0.189±0.01 |
| P+E | 0.558±0.04 | 0.363±0.02 | 0.418±0.03 | 0.317±0.01 | 0.336±0.02 | 0.190±0.01 |
| T+E | **0.601±0.01** | 0.383±0.01 | 0.382±0.05 | 0.314±0.01 | **0.365±0.01** | 0.183±0.01 |
| M+P+T | 0.555±0.02 | 0.388±0.01 | 0.442±0.01 | 0.309±0.01 | 0.366±0.02 | 0.184±0.01 |
| M+P+E | 0.567±0.04 | 0.367±0.02 | 0.419±0.02 | 0.312±0.01 | 0.343±0.03 | 0.191±0.01 |
| M+T+E | 0.596±0.02 | 0.382±0.02 | 0.396±0.06 | 0.320±0.01 | **0.377±0.01** | 0.185±0.01 |
| P+T+E | 0.578±0.01 | 0.373±0.01 | **0.448±0.02** | 0.314±0.01 | 0.360±0.02 | 0.188±0.01 |

**Table S2**. Performance comparison between BLAST and Pseudo2GO in terms of precision, recall and f1-score. We should note that the f1-score used here is different from the one we used in the last section comparing multi-label prediction performance where only top three predictions are considered.

|  | CC | | | MF | | | BP | | |
| --- | --- | --- | --- | --- | --- | --- | --- | --- | --- |
| Method | F1 | P | R | F1 | P | R | F1 | P | R |
| Pseudo2GO | 0.606 | 0.582 | 0.633 | 0.498 | 0.626 | 0.414 | 0.404 | 0.396 | 0.413 |
| BLAST | 0.524 | 0.407 | 0.736 | 0.539 | 0.485 | 0.606 | 0.400 | 0.309 | 0.565 |

**Table S3**. List of GO terms that pseudo2GO predicts with high confidence. For each pseudogene, we calculate the intersection between our top five predicted GO terms and the true annotations. Here are the selected pseudogenes whose predicted top 5 GO terms are all true positives.

| **Gene name** | **MF** | **CC** | **BP** |
| --- | --- | --- | --- |
| PCDHB18P | GO:0005509, GO:0043169, GO:0043167, GO:0005488, GO:0046872 | GO:0016021, GO:0044459, GO:0031226, GO:0044425, GO:0031224 | GO:0007155, GO:0022610 |
| OR8G3P | GO:0038023, GO:0060089, GO:0004984, GO:0004930, GO:0004888 | GO:0005886, GO:0016021, GO:0044425, GO:0016020, GO:0031224 | GO:0007186, GO:0003008, GO:0050789, GO:0050794, GO:0050896 |
| NANOGP1 | GO:0043565, GO:0000976, GO:1990837, GO:0003690, GO:0001067 | GO:0044464, GO:0043229, GO:0044424, GO:0043226, GO:0043231 | GO:2000112, GO:0031326, GO:0050789, GO:0050794, GO:0051252 |
| GOLGA8DP | GO:0005515, GO:0005488 | GO:0044444, GO:0043229, GO:0043227, GO:0043226, GO:0043231 | GO:0009987, GO:0022402, GO:0071840, GO:0006996, GO:0016043 |
| MT1L | GO:0046914, GO:0043169, GO:0043167, GO:0046872, GO:0008270 | GO:0044464, GO:0043229, GO:0044424, GO:0043226, GO:0043231 | GO:0065007, GO:0055080, GO:0048519, GO:0098771, GO:0050896 |
| OR7A2P | GO:0038023, GO:0060089, GO:0004984, GO:0004930, GO:0004888 | GO:0044464, GO:0016021, GO:0044425, GO:0016020, GO:0031224 | GO:0065007, GO:0009987, GO:0007186, GO:0050789, GO:0050794 |
| ZNF137P | GO:0003676, GO:0043167, GO:0005488, GO:1901363, GO:0097159 | GO:0044464, GO:0043229, GO:0044424, GO:0043227, GO:0043226 | GO:0065007, GO:0050789, GO:0050794, GO:0031323, GO:0019222 |
| SSX6P | GO:0003676, GO:0097159, GO:0005488, GO:1901363 | GO:0044464, GO:0043229, GO:0044424, GO:0043227, GO:0043226 | GO:0065007, GO:0080090, GO:0050789, GO:0050794, GO:0019222 |
| SSX9P | GO:0003676, GO:0097159, GO:0005488, GO:1901363 | GO:0044464, GO:0043227, GO:0005634, GO:0043226, GO:0043231 | GO:0010468, GO:0080090, GO:0006355, GO:0050789, GO:0019222 |

**Supplementary figures**


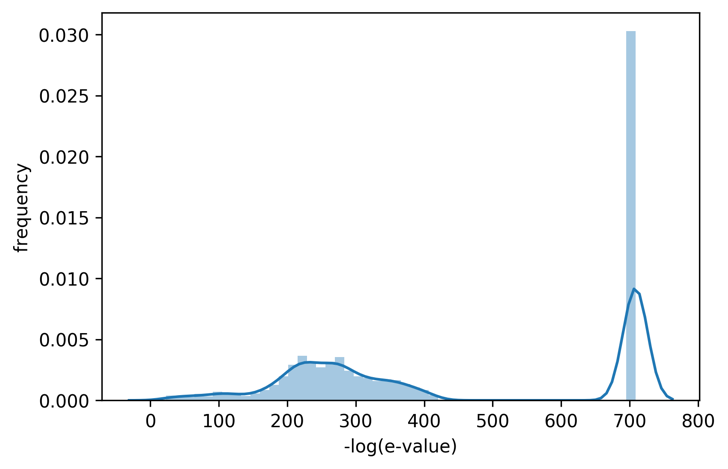


1.
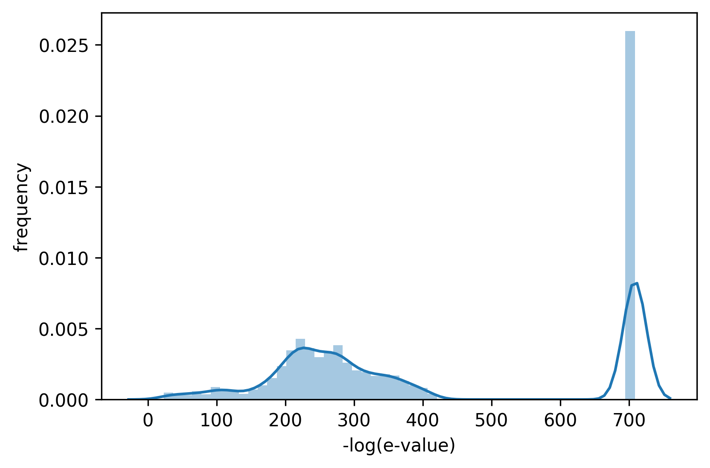
 (b)

**Figure S1**. Distribution of similarity scores between pseudogenes and coding genes. Here we used -log(e-value) as the x-axis. When the e-value equals to zero, we used the minimum float number (2.225e-308) in Python language to represent it, and therefore the corresponding transformed value is around 708. (a): Distribution of similarity scores between 1151 pseudogenes and all other coding genes. (b): Distribution of similarity scores between 1151 pseudogenes and selected 7527 coding genes.


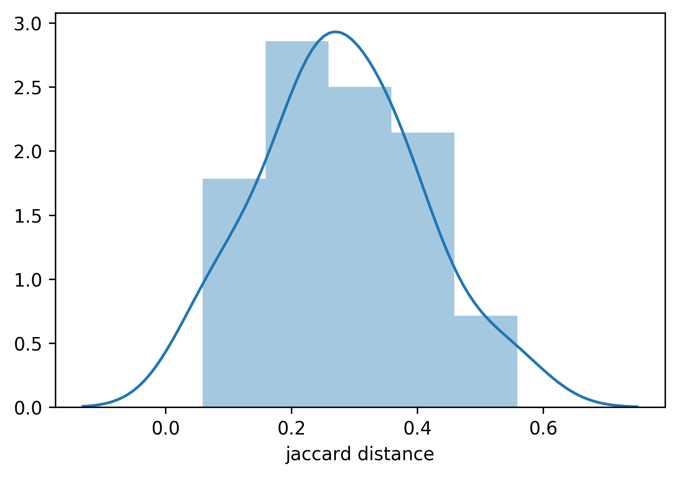

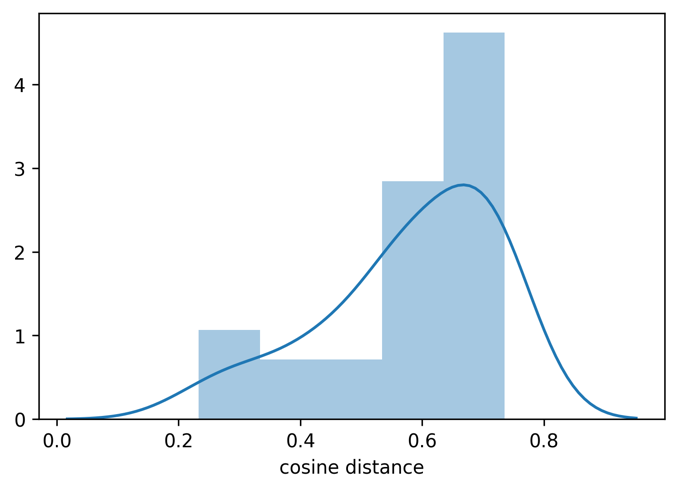

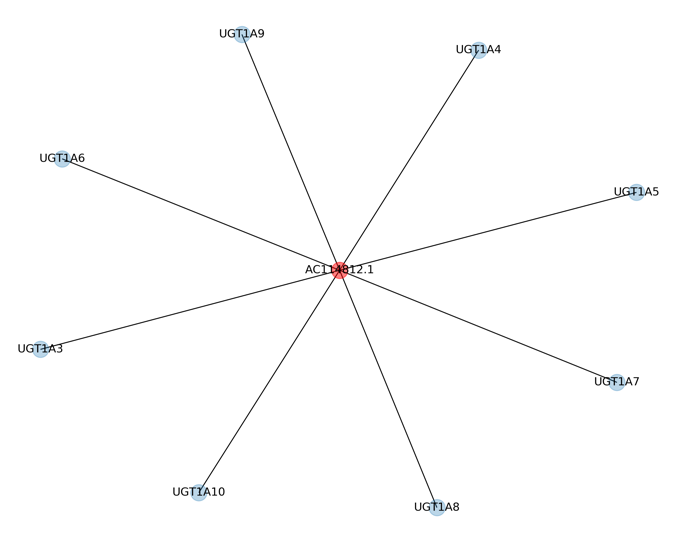


1. (b)

(c)

**Figure S2.** An example when the connecting coding genes of the pseudogene belong to the same paralog family. (a): Local network structure of pseudogene AC114812.1. We only show the pseudogene-coding gene edges while ignoring coding-coding edges in the figure. The 8 neighboring coding genes belong to the same paralog family. (b) Distribution of pairwise node attribute distances among neighboring coding genes. Cosine distance is used to measure the dissimilarity of two node attribute vectors. (c): Distribution of pairwise GO annotation (label) distances among neighboring coding genes. Jaccard distance is used to measure the dissimilarity of two binary label vectors.
